# Biological responses to environmental contamination. How can metal pollution impact signal honesty in avian species?

**DOI:** 10.1002/ece3.4192

**Published:** 2018-07-06

**Authors:** Aneta Dorota Pacyna, Marek Ruman, Jan Mazerski, Żaneta Polkowska

**Affiliations:** ^1^ Faculty of Chemistry Department of Analytical Chemistry Gdansk University of Technology Gdansk Poland; ^2^ Faculty of Earth Sciences University of Silesia Sosnowiec Poland; ^3^ Faculty of Chemistry Department of Pharmaceutical Technology and Biochemistry Gdańsk University of Technology Gdańsk Poland

**Keywords:** carotenoids, feather, melanin, pigments, trace elements

## Abstract

Environmental pollution, for example with metals, can significantly affect the ecosystem balance leading to severe changes. Biologically active pigments are relevant for the appearance and condition of birds. Melanin and carotenoid particles are the most frequently deposited pigments in avian integument. They are responsible for the majority of colors of bird plumage. The phenotypic expression can be affected by metal contamination. It can be manifested as color bleaching or differences in the size of plumage badges. In this study, we performed a comprehensive review of related studies in order to estimate the underlying population effect of this potential dependency. The study is based on the review of the literature regarding several avian species. It was designed to identify an area where the effect of the exposure is still poorly known. The analysis was specifically conducted to investigate the correlation between trace element concentration and eumelanin deposition. Moreover, we searched for factors that could affect spectral properties of feathers with carotenoid‐based pigmentation. As a result, we found carotenoid‐based pigmentation to be of a good use in terms of visual condition assessment. Changes in melanin‐based pattern should be analyzed separately for eu‐ and pheomelanin as well as for a range of essential and toxic elements. Comprehensive studies on the subject are still scarce. Therefore, the issue requires further investigation.

## 1 INTRODUCTION

Plumage coloration is used by birds to communicate signal honesty, informing on the individual's quality status (Bortolotti, Blas, Negro, & Tella, 2006; Gunderson, Frame, Swaddle, & Forsyth, 2008; McGraw, Hill, & Parker, 2005; Zahavi, 1975). Signals must be costly to be reliable, otherwise “low‐quality” individuals would easily benefit from faking (Zahavi, 1975; Olson and Owens, 1998). The ability to sequester certain minerals within melanin‐ or carotenoid‐based parts can be used to trace dietary access to such elements. Pigment‐based feather coloration can be also used to track the ability of individuals to cope with environmental and physiological challenges in gaining pigments (McGraw, 2005).

Melanin‐ and carotenoid‐based plumage pigmentation is widespread across avian species. Numerous studies have been dedicated to enhancing knowledge about its origin, synthesis, and factors affecting its expression (e.g., McGraw, 2005; Niecke, Heid, & Krüger, 1999; Niecke, Rothlaender, & Roulin, 2003). However, information on many aspects of the effect of environmental factors such as metal pollution on trait display is still scarce. The knowledge gap is especially evident in respect to studies focusing on the correlation between trace element concentration and pigment deposition.

Enrichment with several elements was determined in melanized parts of feathers. Some studies clearly showed calcium and zinc enrichment within pigmented spots (e.g., Niecke et al., 1999, 2003). According to the study by Dauwe and Eens (2008), metal pollution, in particular lead, cadmium, copper, and zinc, enhanced the expression of the melanin‐based breast stripe in great tits (*Parus major*) from the most polluted sites. At the same time, yellow carotenoid coloration was negatively correlated with metal contamination. Black sections of the feather shaft were significantly enriched with zinc, calcium, copper, and magnesium according to a study by Hanć, Zduniak, Erciyas‐Yavuz, Sajnóg, and Barałkiewicz (2017). In Giraudeau, Mateos‐Gonzalez, et al. (2015), the determined total content of metals was positively correlated with the size of the melanin‐based black tie. Melanin pigmentation was also positively correlated with copper level and negatively with chromium concentration (Giraudeau, Mateos‐Gonzalez, et al., 2015).

Melanin is synthesized in the body either from tyrosine or from phenylalanine as the end product of the biochemical pathway (Hearing, 1993). Metal ions can modulate the activity of the enzyme tyrosinase that catalyzes melanin production (McGraw, Beebee, Hill, & Parker, 2003; Zduniak, Surmacki, Erciyas‐Yavuz, Chudzińska, & Barałkiewicz, 2014). Melanin carboxyl groups can also bind metal ions and serve as cation chelators facilitating body detoxification (Chatelain, Gasparini, Jacquin, & Frantz, 2014; Dauwe, Bervoets, Pinxten, Blust, & Eens, 2003; Zduniak et al., 2014). Because melanin production is associated with significant cost, it is assumed that only the healthiest individuals would be capable of producing the darkest version of a melanin‐based color trait (Meunier, Figueiredo Pinto, Burri, & Roulin, 2011). For some species, however, for example, females of the common eider (*Somateria mollissima*)*,* pale coloration could be more costly to produce than dark coloration (Hanssen, Folstad, & Erikstad, 2006). In such a situation, the trait displaying cost could be misleadingly interpreted.

In contrast to melanin, vertebrates and insects cannot synthesize carotenoids de novo, and pigment particles need to be acquired from food sources (Goodwin, 1984; Sillanpää, Salminen, Lehikoinen, Toivonen, & Eeva, 2008). Carotenoid uptake from food is limited by the species physiology and genotype (Olson and Owens, 1998). The presence of carotenoid is relevant for the condition of birds due to its role in both health maintenance and ornamental signaling (Tummeleht, Mägi, Kilgas, Mänd, & Hõrak, 2006). More colorful plumage can indicate better body condition and better ability to assimilate carotenoids from food during molt (Giraudeau, Chavez, Toomey, & McGraw, 2015). Metals can affect the abundance and quality of carotenoid‐rich food sources (Eeva, Lehikoinen, & Rönkä, 1998; Eeva et al., 2008; Geens, Dauwe, & Eens, 2009). Also, birds from more polluted places are commonly believed to use more carotenoids for antioxidant defense to reduce oxidative stress levels, which leaves less to be used for feather coloration (Geens et al., 2009).

This study focuses primarily on potential effects of trace elements on melanin‐ and carotenoid‐based coloration and therefore signal quality. Melanin‐based coloration has been believed to be controlled exclusively genetically for many years (Niecke et al., 2003). Recent findings, however, confirm the hypothesis that both carotenoid (Eeva et al., 2008) and melanin deposition is condition‐dependent (Guindre‐Parker & Love, 2014). This study presents a brief review of the limited existing evidence of a link between environmental contamination and pigment deposition in bird feathers.

## 2 METHODS

### 2.1 Data sources

The study is based on the extensive search of literature concerning the associations between chemical element concentration and pigment deposition measured in feathers. Keyword searches were performed, particularly by means of Web of Science, Google Scholar, and Wiley database. The data search was last updated in October 2017. The following keyword combinations were used: “metal, element analysis, melanin ornaments,” “feather, metal, melanin,” “bird, melanin coloration,” “feather, carotenoids,” “bird, carotenoid coloration,” and “feather, carotenoids, metal”. A forward search was also performed for articles cited in papers such as McGraw (2003), Eeva et al. (2008) and for abstracts, book chapters, and conference papers covering the topic. We also searched for previous reviews focusing on melanin‐ and carotenoid‐based coloration and articles cited in them (McGraw, 2008; Santos et al. 2011, Meunier et al., 2011). In total, about 70 papers were found, screened by abstract or full text. Due to their limited availability, we do not use unpublished studies in the paper to avoid charges of a publication bias (known as the “file‐drawer” problem, Rosenthal, 1979). Only studies published in English were included.

Because carotenoid pigmentation is widely represented among multiple species, we searched for papers concerning metals as a factor affecting carotenoid‐based coloration. Due to differences in pigment acquisition as compared to melanin, we focused on food chain disturbances resulting in potential color bleaching.

### 2.2 Average effect size

**Figure 1:**
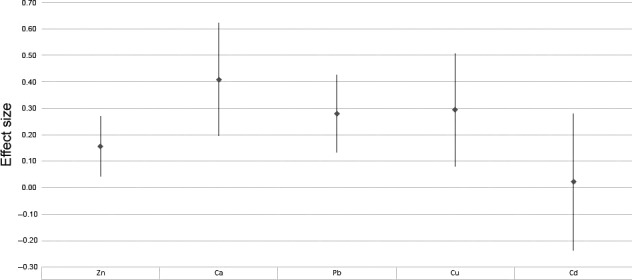
Forest plot of the effect size of correlation between the element concentration and the eumelanin deposition (based on *r*)

We calculated an overall effect size for the correlation between concentrations of several trace elements in feathers and eumelanin deposition based on original studies performed on eight species. Comprehensive studies with simultaneous quantity determination of color and trace element concentration are still scarce; thus, we restricted the meta‐analysis to eumelanin. Because the data were incomplete, all trace elements (zinc, lead, cadmium, calcium, copper) were examined separately (Table 1; Figure 1). In analysis, following studies were used: Chatelain et al., 2014; Chatelain, Gasparini, & Frantz, 2016; Giraudeau, Chavez, et al., 2015; Giraudeau, Mateos‐Gonzalez, et al., 2015; Gochfeld et al. 1991; McGraw, 2007; Niecke et al., 2003; Zduniak et al., 2014 . Small sample sizes in the available research result in a high confidence interval. In the majority of cases, the relationships between variables were presented as correlation coefficient *r*. If the relationship was presented in the form of significance level *p*, and assuming that the correlation significance was tested using a *t*‐test, the value was recalculated for *r*, according to the following formula (Rosenthal & Rubin, 2003):

$$r=\sqrt{\frac{t^{2}\left(p\right)}{t^{2}\left(p\right)+\left(N-2\right)}}$$

**Table 1:** Results of the meta‐analysis of the association between trace element concentration and eumelanin deposition in feathers

Metal	Mean effect size *r* (*Zr*)	The standard error *r* (*Zr*)	The lower limit of the confidence interval for *r* (*Zr*)	The upper limit of the confidence interval for *r* (*Zr*)	*p*	Sample size (number of birds)
Zinc	0.156 (0.163)	0.058 (0.058)	0.270 (0.282)	0.041 (0.043)	0.0038 (0.0069)	283
Calcium	0.408 (0.428)	0.109 (0.132)	0.623 (0.693)	0.195 (0.163)	0.00009 (0.0019)	60
Copper	0.294 (0.308)	0.109 (0.108)	0.508 (0.549)	0.080 (0.068)	0.0035 (0.012)	72
Lead	0.280 (0.292)	0.075 (0.074)	0.426 (0.475)	0.133 (0.108)	0.00009 (0.0019)	122
Cadmium	0.022 (−0.009)	0.132 (0.132)	0.281 (0.255)	−0.238 (−0.275)	0.4343 (0.942)	60

*Note*

Presented average effect sizes were calculated using correlation coefficient *r* and its normalizing transformation Fisher's Z: *Z*(*r*) (details in Method section).

where: *t*(*p*)—Student's *t* statistic for significant level *p* and degrees of freedom equal to *N*‐2.

We performed a fixed effects meta‐analysis using correlation coefficient *r* and its normalizing transformation Fisher's Z: *Z*(*r*) to estimate the mean effect size. Effect sizes based on correlation coefficient *r* were calculated based on Olkin‐Pratt (DSL) fixed‐effect meta‐analytical approach, as described by Schulze (2004) in R environment (Lumley, 2012 (“rmeta”package); R Core Team, 2017) and Fisher's Z: *Z*(*r*) were calculated in Excel (Excel, 2010).

When correlation coefficients were recalculated for its normalizing transformation Fisher's Z: *Z*(*r*), the following formula was used:

$$Z\left(r\right)=\frac{1}{2}\text{ln}\frac{1+r}{1-r}=\text{arctanh}\left(r\right)$$

If variable *X* and *Y* had a combined two‐dimensional normal distribution, and if the individual pairs (*X*_*i*_, *Y*_*i*_) were independent, then *Z*(*r*) had approximately normal distribution with a mean:

$$m_{Z}=\frac{1}{2}\text{ln}\frac{1+\rho }{1-\rho }$$

and a standard deviation:

$$s_{Z}=\frac{1}{\sqrt{N-3}}$$

## 3 RESULTS AND DISCUSSION

### 3.1 Melanin coloration

The dependency between metal abundance and pigment expression has been rarely subject to experimental or field studies. This study compiles available research from a collection of empirical studies to estimate the variance of the underlying population effect of this potential dependency. Melanin is synthesized from amino acid precursors within melanocytes (Michalik et al. 2010, McGraw et al., 2003). Its production can be costly for the bird in terms of energy and time involved (Gunderson et al., 2008). In vertebrates, melanin takes two main forms: eumelanin which gives darker black, brown, or gray color, and pheomelanin which gives reddish or buff color (McGraw, Safran, & Wakamatsu, 2005). Elements such as Ca, Cu, Fe, and Zn are necessary in the process of intermediate products formation in syntheses of both melanin types (McGraw et al., 2003). They also play an indispensable role in multiple physiological body functions (Bogden & Klevay, 2000). Therefore, their level could be a consequence of their baseline distribution in the entire body (Zduniak et al., 2014). Due to a vast diversity of integument coloration patterns and multiple factors affecting deposition, it is not fully clear how metal distribution is shaped within melanized parts. Here, we calculated the average effect size of concentrations of five chemical elements and eumelanin deposition in spotted parts of feather. Due to the limited number of studies, however, the analysis can only be treated as support for further studies. The most significant effect size was recorded for calcium (Table 1). Calcium is relevant in many physiological processes, including skeletal mineralization (Zduniak et al., 2014). It is essential for the survival of birds. It induces aggregation of melanin particles, making feather more durable and resistant to mechanical stress (Niecke et al., 1999). Synchrotron X‐ray analyses showed that the distribution of calcium is controlled by melanin pigment and is nonuniformly distributed throughout feather parts (Edwards et al., 2016; Howell et al., 2017). In a study performed on male Barn Owls, Roulin, Dauwe, Blust, Eens, and Beaud (2006) demonstrated a positive relationship between enhanced calcium deposition in humerus bone and plumage spottiness. This study provided no evidence of the production of eumelanin pigment generating additional cost for the organism in terms of extra calcium use (Roulin et al., 2006).

In an experimental study by McGraw (2007), the black breast eumelanin‐based patch in male zebra finches (*Taeniopygia guttata*) increased its size after calcium supplementation. Therefore, especially in the case of species with a hierarchy linked to the size of melanin‐based patches, calcium availability could be expected to be a factor affecting signal honesty.

Results regarding zinc were not homogenous for all studies (*Q* = 8.940, χ^2**^ *=* ^**^7.815 for α = 0.05, 3 degrees of freedom; *p* = 0.03), and the overall size effect was not significant (*p* = 0.081). Zinc is an essential trace element for the physiological condition of birds, and its distribution might be focused in a repetitious banding pattern (Howell et al., 2017). Niecke et al. (2003) found zinc enhancement within spotted feathers of the barn owl (*Tyto alba*). The relationship was noticeably different for eumelanized parts (*r* = 0.18, *p* = 0.26) and reddish brown parts with pheomelanin (*r* = −0.72, *p* < 0.001). Zinc concentration was 2.7 times higher within black spots, compared to unspotted parts. It was also found in nonpigmented feather parts. Zinc appeared to have higher affinity for pheomelanin than eumelanin, and relative, not absolute concentrations correlating with pigment patterns (Edwards et al., 2016).

Similarly to zinc and calcium, copper is controlled by melanin pigment patterns in feathers (Edwards et al., 2016). Both analyses in this study employed a similar sample size in the calculation of average effect size. The values of correlation coefficients, however, significantly differed from each other. The data were homogenous as *Q = *2.671, χ^2^ = 3.841 for α = 0.05 and one degree of freedom, *p* = 0.102. The overall effect size was statistically significant (Table 1). Macro‐ and microminerals are scarce in the diet of many bird species (Zduniak et al., 2014). Deposition of valuable elements such as calcium, zinc, or copper in inert dead tissue enables their further usage. However, as shown in the study by Roulin et al. (2006), eumelanism may be compromised against other physiological processes such as calcium storage in bones that requires the same essential elements. Eumelanin pigmentation may therefore reflect the physiological ability to absorb and store elements in body parts (Roulin et al., 2006).

Elevated levels of toxic compounds were also found in melanized feather parts. Regarding analysis of average effect size for lead, only one of the studies (Chatelain et al., 2016) showed a statistically significant relationship. The remaining studies provided no unambiguous results, probably due to a small sample size. Data homogeneity was confirmed as *Q = *4.412, χ^2^ = 7.815 for α = 0.05; 3 degrees of freedom, *p *=* *0.220. The overall effect size for all studies was statistically significant. In the case of cadmium, results of only one study including three different species were found. Data homogeneity was confirmed (*Q = *1.012*,* χ^2^ = 5.991 for α = 0.05 and 2‐degrees of freedom, *p* = 0.603). The overall effect size was not statistically significant.

Dauwe and Eens (2008) found that lead, cadmium, copper, and zinc enhanced the expression of the melanin‐based breast stripe in great tits (*Parus major*). In contrast to zinc, Chatelain et al. (2014), found no correlation between lead concentration and melanin‐based coloration score in urban feral pigeons after 1 year of captivity. Because environmental factors may have hidden the correlation pattern during field studies, experimental research on domesticated species would help to understand the adaptive function of element deposition in melanized parts. The ability to cope with metals after they exceed toxic levels can be used to signal condition of an individual bird (McGraw, 2003). Knowing the pattern and effect of toxic metal deposition in melanized parts of feathers on signal honesty may shed some light on birds adaptation abilities in high polluted places.

### 3.2 Carotenoids

Carotenoid deposition is most likely species‐dependent. It is influenced by the abundance and quality of carotenoid‐rich food, as well as the bird's ability to assimilate pigments. Giraudeau, Mateos‐Gonzalez, et al. (2015) show a negative correlation of carotenoid‐based coloration with mercury concentration (*p* = 0.02), as well as with the sum of metals (Hg, Cu, Pb, Cr, As, Cd, Sb, and Zn) (*F*_1,30 _= 2.07, *p* = 0.16). Chicks of great tits living nearer a pollution source (a factory complex producing Cu, Ni, and fertilizers) had paler yellow plumage than chicks living further away from the factory (Eeva et al., 1998). A study by Geens et al. (2009) shows similar results. Giraudeau, Chavez, et al. (2015) confirmed the finding in reference to house finches (*Haemorhous mexicanus*), evidencing that urban birds had paler plumage than desert birds at capture. Carotenoid composition and distribution in feathers are affected by genetic, metabolic, physiological, and dietary factors (Brush & Power, 1976). In more polluted areas, a different type of prey would be eaten, with a predominance of less carotenoid‐rich food sources, for example, spiders instead of caterpillars (Geens et al., 2009; Koivula, Kanerva, Salminen, Nikinmaa, & Eeva, 2011). Many insect species can selectively accumulate more carotenoids such as lutein than others (Ahmad, 1992; Sillanpää et al., 2008). Some birds can also have this ability (McGraw et al., 2003).

In more polluted areas, plants could contain less carotenoid pigments due to elevated oxidative stress levels. Therefore, caterpillars feeding on them would also be of lower quality (Isaksson & Andersson, 2007). This explanation, however, is not always reflected in studies. In the study by Sillanpää et al. (2008), insects had higher body carotenoid concentration when derived from more polluted places, or there was no association between polluted and unpolluted areas.

Isaksson and Andersson (2007) found that caterpillars living in the urban environment were more abundant and heavier in comparison with the rural population, although they also contained less carotenoids in their bodies. Chicks from urban areas were fed approximately twice more often by their parents, providing carotenoid availability similar to that of rural nestlings.

Coloration often increases in individuals with age (Delhey & Kempenaers, 2006). Less colorful individuals have less chance to survive until adulthood (Pagani‐Nuñez & Senar, 2012). The estimation of the carotenoid content and search for carotenoid‐rich food sources requires experience, not available to young birds at the beginning. Younger birds can be also more affected by parasitism and higher levels of oxidative stress; therefore, more carotenoids might need to be used as antioxidants (Geens et al., 2009; Giraudeau, Barcelo, & Senar, 2014). The ability of younger and older birds to extract and assimilate carotenoids from food may also differ (McGraw & Parker, 2006). Terms of molting and seasonal differences in food abundance and quality, as well as other dietary antioxidants protecting carotenoids from oxidation and bleaching (Del Val, Quesada, & Senar, 2010; Giraudeau et al., 2014), can also be responsible for major differences between age classes. Carotenoid‐based coloration could possibly reflect only the level of other antioxidants, such as vitamin A, acting against oxidative bleaching of carotenoids (Constantini & Møller, 2008; Hartley & Kennedy, 2004) .

### 3.3 Knowledge gaps and research needs

Feathers are complex biological structures that display a diversity of pigmentation patterns. Melanin bounds many compounds and retains them for long periods of time. Therefore, melanin‐based pigmentation could be used to assess long‐term physical condition of the individual (Hung & Li, 2015). Melanin coloration results from the co‐occurrence of both eumelanin and pheomelanin pigments at different concentrations (McGraw & Parker, 2006; Zduniak et al., 2014). Differences in their bounding capacities may explain the distinct spatial distribution pattern across species for some of the elements (Howell et al., 2017). The ability of pheomelanin to store elements is still largely understudied. Only a few examples can be found in the literature (Niecke et al., 2003; Zduniak et al., 2014).

A huge diversity of within‐feather pigmentation patterns exist. The mechanisms determining pigment distribution into keratinocytes are of interest to scientists (Prum & Williamson, 2002). Little is known, however, about the fundamental biological processes leading to orderly patterns and integumental tissue development. It seems that highly melanic birds should be favored in an environment with a high level of certain metals (Chatelain et al., 2014). Many birds which adapt to live in European urban areas have melanin‐based plumage coloration (Chatelain et al., 2016). Yet, the mechanism and effect of toxic metals deposition are significantly understudied.

Both carotenoid‐ and melanin‐based pigmentation is considered a way of signaling which is costly in production. Production of eumelanin is linked to a major physiological process, namely skeletal mineralization (Roulin et al., 2006). Carotenoid deposition is most likely species‐dependent and is influenced by the abundance and quality of carotenoid‐rich food, as well as the bird's ability to assimilate pigments.

Metal ions can affect the pigment synthesis pathway. The degree of melanin and carotenoid deposition, however, is not necessarily clearly linked to honest signaling, because additional environmental and physiological factors might correspond to it. The profound understanding of the issue requires further research concerning free‐living birds, domesticated, and captivated species. They would show different pigment acquisition.

## 4 CONCLUSION

New selection pressure caused by anthropogenic activity challenges the ability of birds to adapt and survive in moderately and strongly polluted habitats (Giraudeau, Chavez, et al., 2015). In the environment subject to transformations, animals have to adapt to new conditions and xenobiotic influence. The impact of trace elements on biota is intensively investigated. Several empirical studies showed that melanin bounding capacities may allow some body detoxification. Essential and nonessential elements, however, behaved differently and should be treated separately. Carotenoid‐based color bleaching is a first visual signal for mates and rivals providing information on the individual's ability to cope with environmental and physiological challenges. More data are still required, however, on many aspects facilitating the understanding of the mechanisms responsible for plumage coloration and the actual cost of pigment deposition.

## CONFLICT OF INTEREST

None declared.

## AUTHOR CONTRIBUTION

ADP was responsible for concept development, literature search, work design, and manuscript writing. MR and ZP provided text corrections, and JM provided the meta‐analysis. All of the authors took part in the data interpretation and critical draft revision and contributed to editing the manuscript. All authors read and approved the final manuscript.
